# Coding of medically unexplained symptoms and somatoform disorders by general practitioners – an exploratory focus group study

**DOI:** 10.1186/s12875-018-0812-8

**Published:** 2018-07-27

**Authors:** N. J. Pohontsch, T. Zimmermann, C. Jonas, M. Lehmann, B. Löwe, M. Scherer

**Affiliations:** 1 Department of General Practice / Primary Care, University Medical Center Hamburg-Eppendorf, Martinistr. 52, 20246 Hamburg, Germany; 2 Department of Psychosomatic Medicine and Psychotherapy, University Medical Center Hamburg-Eppendorf and Schön Clinic Hamburg Eilbek, Martinistr. 52, 20246 Hamburg, Germany

**Keywords:** ICD, General practitioner, Germany, Focus group, Somatoform disorder, Medically unexplained symptoms, Qualitative research

## Abstract

**Background:**

Medically unexplained symptoms (MUS) and somatoform disorders are common in general practices, but there is evidence that general practitioners (GPs) rarely use these codes. Assuming that correct classification and coding of symptoms and diseases are important for adequate management and treatment, insights into these processes could reveal problematic areas and possible solutions. Our study aims at exploring general practitioners’ views on coding and reasons for not coding MUS/somatoform disorders.

**Methods:**

We invited GPs to participate in six focus groups (*N* = 42). Patient vignettes and a semi-structured guideline were used by two moderators to facilitate the discussions. Recordings were transcribed verbatim. Two researchers analyzed the data using structuring content analysis with deductive and inductive category building.

**Results:**

Three main categories turned out to be most relevant. For category a) “benefits of coding” GPs described that coding is seen as being done for reimbursement purposes and is not necessarily linked to the content of their reference files for a specific patient. Others reported to code specific diagnoses only if longer consultations to explore psychosomatic symptoms or psychotherapy are intended to be billed. Reasons for b) “restrained coding” were attempting to protect the patient from stigma through certain diagnoses and the preference for tentative diagnoses and functional coding. Some GPs admitted to c) “code inaccurately” attributing this to insufficient knowledge of ICD-10-criteria, time constraints or using “rules of thumb” for coding.

**Conclusions:**

There seem to be challenges in the process of coding of MUS and somatoform disorders, but GPs appear not to contest the patients’ suffering and accept uncertainty (about diagnoses) as an elementary part of their work. From GPs’ points of view ICD-10-coding does not appear to be a necessary requirement for treating patients and coding might be avoided to protect the patients from stigma and other negative consequences. Our findings supply a possible explanation for the commonly seen difference between routine and epidemiological data. The recent developments in the DSM-5 and the upcoming ICD-11 will supposedly change acceptance and handling of these diagnoses for GPs and patients. Either way, consequences for GPs’ diagnosing and coding behavior are not yet foreseeable.

**Electronic supplementary material:**

The online version of this article (10.1186/s12875-018-0812-8) contains supplementary material, which is available to authorized users.

## Background

GPs are usually confronted with a patient population presenting (unspecific) symptoms and unselected health care problems [1] and patients with medically unexplained symptoms (MUS) are numerous in primary care [2, 3]. Up to 20% of patients visiting the GP suffer from somatic symptoms without a sufficient organic explanation [4, 5]. There are many terms used apart from MUS, like “bodily distress syndrome” [6], “functional symptoms” and “somatic symptom distress” [7, 8] and critique about the ambiguity and the pejorative connotation of the term MUS is often expressed [9]. Anyway, this term was chosen as it is widely used in research literature and corresponds to the definition of somatoform disorders in the International Classification of Diseases, 10. Revision (ICD-10, [10]) and Diagnostic and Statistical Manual of Mental Disorders (DSM) IV [11], albeit the discussion is ongoing and fueled by a great diverseness of research literature [12, 13]. Not all patients with (severe) MUS will eventually be diagnosed with a psychiatric somatization disorder (ICD-10 F45.x), as preferences for diagnoses differ according to the specialty [14]; medical specialties prefer functional somatic syndromes [6] like fibromyalgia, chronic fatigue syndrome and chronic benign pain syndrome [15]. There is evidence that coding might be based upon the specialty (e.g., somatic or psychiatric) of the treating physician [16] and might differ between primary and secondary care [17]. The classification of somatoform disorders has been revised in the past [8] and will experience further revisions with the publication of the ICD-11 [18].

In health care systems all over the world it is mandatory to record a patient’s condition (symptoms or diseases) by coding it according to different coding systems, for example, via the ICD-10 [10] or the International Classification of Primary Care (ICPC 2-PLUS, [19]) both in primary and secondary care. It is widely considered indispensable to code with high validity and integrity to deliver high quality, efficient and safe care for patients [13, 20]. The underlying assumption that better coding of information results in better care for patients [21–23] is not challenged very often despite not being clearly proven. Reliable data on incidence and prevalence of diseases are also important for research purposes. Researcher need reliable data on the morbidity of a population to, for example, design and evaluate health care interventions, evaluate quality of care and monitor changes in longitudinal studies [24–26]. Other purposes are epidemiology, outcome studies and the identification of suitable individuals to participate in clinical trials [27]. In contrast, studies show that the reliability of coded diseases in primary [24, 28–30] and secondary [26, 31] health care settings is often poor, including for example the phenomenon of “billing diagnoses” [24] which have their origin in the German Medicinal Products Directive and are used to legitimate certain prescriptions. Furthermore epidemiological data and routine data on functional and somatoform disorders are not congruent with each other [16].

In Germany it is mandatory to code all reasons for consultations and diagnoses for which a contracted physician performs diagnostic or treatment services in an ambulatory setting according to the ICD-10-GM (German Modification, [32]). As a result, diagnostic or treating efforts cannot be reimbursed without a legitimate diagnosis. Four-digit-coding (e.g., F45.9) is sufficient for German general practitioners (GPs), but it is mandatory to distinguish between “tentative diagnosis”, “(asymptomatic) condition after a confirmed diagnosis”, “excluded diagnosis” and “confirmed diagnosis”. In Austria the ICD-10 is also used in primary care. Other countries like for example Belgium, Denmark, France and many more use the ICD-10 in secondary care, but the ICPC, a system to classify reasons for encounters, problems/diagnosis managed and interventions performed, in primary care [33].

The ICD-10 allows “double-coding” according to etiology and localization of diseases. That means, for example, the same clinical phenomenology without distinct medical explanation (e.g., MUS) can be coded using organ-related codes as a functional disorder (e.g., K58.0 irritable bowel syndrome) or as mental disorders using codes from chapter F on mental and behavioural disorders (e.g., F45.32 somatoform autonomic dysfunction lower gastrointestinal tract) [16]. Patients with MUS having primarily physical rather than psychological symptoms usually consult general practitioners rather than mental health care specialists first [8], however, somatoform categories are used less often by general practitioners [8] and other clinicians (underassignment, p. 143 [34]). Diagnoses not adequately adhering to guidelines or diagnostic frameworks may hinder communication between primary and specialized care [17] and lead to inadequate treatment [34]. Despite that, one may assume that patients do receive treatment even without a diagnosis of a somatoform disorder, since the management of unspecific symptoms is a core competency of general practitioners [1].

General practice is characterized by a (long-term) social interaction between a patient and the treating general practitioner and characterized by uncertain diagnoses [35] while the act of coding implies a diagnostic certainty, which is hardly obtained with MUS or somatoform disorders. Scobie et al. [36] found that mental and psychological morbidities were coded less often as GPs reported them to be difficult to code. In addition, many health care systems seem to be biased; tolerating the under- and misdiagnosis of mental disorders, while deeming it unacceptable to overlook a physical diagnosis [36]. Not all GPs deem the classification of diseases as (very) important in respect to the care of patients [26]. Conditions with obvious diagnostic features have a higher rate of recording than conditions with more subjective criteria [37]. There is evidence that GPs rarely code somatization syndromes in daily practice [8, 38]. Not only patients fear stigmatization through psychiatric diagnoses [8, 39], but GPs could also refrain from recording “stigmatizing” diagnosis to protect the patient from suffering negative societal and economic consequences [8, 39].

The reasons for not recording MUS/somatoform disorders are not clear yet [34] and need to be evaluated in qualitative research designs. There is a lack of studies on German GPs’ coding behavior especially in respect to MUS or somatoform disorders. Assuming that correct classification and coding of symptoms and diseases are an important stepping stone for adequate management and treatment, and essential for research purposes, insights into these processes could help identifying problematic areas and possible solutions. Therefore, our study aims to explore German GPs’ views on and approaches to coding and reasons for non-recording of MUS or somatoform disorders.

## Methods

This study is part of the project “Identification of barriers and difficulties involved in the process of diagnosing somatic symptom disorders in primary care”. The project incorporates three studies, 1) focus groups with GPs, 2) interviews with affected patients and their treating GPs and 3) a survey with GPs [40]. This article presents results from the focus group study. The project was funded by the German Research Foundation (DFG; funding numbers SCHE 1689/5–1, AL 1459/5–1 and LO 766/13–1) and registered at the German Clinical Trial Register under the ID: DRKS00009736.

### Ethics

Ethics approval was obtained from the Ethics Committee of the Hamburg Medical Association (Germany) on April 7th, 2015 (approval number PV4763). All participants received oral and written information, had the possibility to ask further questions and gave their informed consent for the focus groups to be recorded, the recordings to be transcribed verbatim and the results of the analysis to be published anonymously.

### Researcher characteristics

Focus groups were moderated by TZ, CJ and NJP. Data was analyzed by CJ and NJP. TZ is a male trained psychologist and postdoctorate researcher (exemplary research areas: mental disorders in primary care, quantitative research). He is experienced in moderating workshops and group discussions. CJ is a female GP registrar and postdoctorate researcher. Referring to research she was new to the field of psychosomatics, but has additional qualifications in basic psychosomatic care (“Psychosomatische Grundversorgung”). She was a first-time moderator and analyzer, but received training on moderating focus groups and qualitative content analysis prior to data collection and analysis. NJP is a female trained psychologist and postdoctorate researcher (exemplary research areas: potentially inadequate medication in primary care, rehabilitation, quality indicators), with comprehensive experience in conducting focus groups and interviews as well as qualitative data analysis. ML is a male trained psychologist and postdoctorate researcher, BL is a full professor of Medicine, board certified both in Psychosomatic Medicine and Internal Medicine and trained as psychologist and psychotherapist, MS is a full professor of Medicine, board certified in General Medicine.

### Recruitment (pilot group)

We invited 20 members of the German College of General Practitioners and Family Physicians (DEGAM - Deutsche Gesellschaft für Allgemeinmedizin und Familienmedizin; general practitioners also working in research with varying degrees of specialized training and years of practice from all over Germany) via e-mail to participate in the pilot focus group. The e-mailed invitation included a short study description and an invitation, as well as detailed study information granting an allowance.

### Recruitment (main study)

We followed a purposive sampling approach [41]. To recruit participants for the five focus groups conducted in Hamburg, a random sample of general practitioners registered in the Hamburg metropolitan area (*N* = 500) and adjacent counties of Lower Saxony and Schleswig-Holstein (Stade, Harburg, Pinneberg, Segeberg, Stormarn and Herzogtum Lauenburg, *N* = 500) were invited via mail to participate in the focus groups. The sample was drawn from a register of 2313 GPs (Hamburg *N* = 1343, adjacent counties *N* = 970) maintained by the Institute of Primary Medical Care of the UKE. Invitation letters consisted of (1.) a short study description and invitation, (2.) a response sheet, (3.) detailed study information (communicating goals of the study), and (4.) a short demographic questionnaire. The invitation granted an allowance and four credit points for vocational training by the Medical Association of Hamburg.

Aiming at conducting five heterogeneous groups in respect to GPs’ gender, years of practice (< 10 years vs. > 10 years) and area of registration (HH or adjacent county) comprised of around eight participants per group, we determined the need to invite 1000 GPs expecting a response rate of 5%. We received feedback from 141 GPs, of which 81 were willing to participate in the study. 82 letters were returned to sender due to either an unknown new address (*N* = 74), the practice being closed (*N* = 6) or the addressee being deceased (*N* = 2).

### Data collection (pilot group/main study)

The pilot focus group was moderated by TZ and NJP following a semi-structured guideline (see below). The discussion lasted 123 min. All other focus groups were moderated by CJ and NJP using the slightly revised semi-structured guideline. Discussions lasted between 107 and 114 min (Ø 111 min). The discussions were digitally recorded in full length and logged by a trained student research assistant.

### Vignette development

Five different vignettes were used to provide a well-defined stimulus for the focus groups. The vignettes for focus groups were derived from anonymized and modified cases from the outpatient department of psychosomatics at the University Medical Center Hamburg-Eppendorf. The story was set in a primary care practice. The first half of the vignette outlined the relationship between the patient and the GP. The main complaints were presented as well as some background information on psychosocial circumstances. Part one also provided a résumé of former diagnoses, results of physical examinations, laboratory findings or other diagnostic results. The second half of the vignette plotted another consultation by the patient. The complaints would have evolved, mostly in an unfavorable way. The patient’s attitude towards the physician’s behaviour, e.g. suggesting a referral to a specialist or introducing a bio-psycho-social disease model, was described. Vignettes contained some possible strategies on dealing with somatization to maintain authenticity whilst avoiding narrowing the options of what to do. Vignettes were varied in different aspects. These included variation of sex, age group, main type of bodily complaints, ICD 10-codes that match best with the description given, fulfillment of the new diagnostic criterions of DSM-5 somatic symptom disorder and corresponding DSM-IV disorders, and the number of symptoms and diagnostic barriers that were implicitly embodied in the story.

### Focus group guideline

The focus group guideline was structured into four main sections. Each group discussion started with the presentation of a patient vignette to be discussed. Vignettes are “short stories about hypothetical characters […], to whose situation the interviewee is invited to respond” (p. 105, [42]). Vignettes can act as an icebreaker or warm-up exercise in focus groups to get the discussion between the participants started [43]. After presenting the first part of the vignette, moderators stimulated discussion by asking: 1) Which problems/difficulties do you face at this point? 2) What would you do with this patient?. After the presentation of the second part of the vignette, moderators asked: 1) Which problems/difficulties do you face now? 2) What impedes diagnosing a somatoform disorder for this patient? 3) How do you recognize such a disorder? 4) How do you document such a disorder?.

The vignette discussion was followed by a discussion of barriers to and helpful tools for diagnosing somatoform disorders, the coding of diagnoses and the changes in the DSM-5 (somatic symptom disorder). See Additional file 1 for questions relevant for the present study. The semi-structured form of the guideline allowed for questions deviating from the guideline topics, if unexpected important aspects were mentioned or if the participants’ accounts needed more clarification. Piloting the guideline resulted only in minor changes in the wording of some questions.

### Protection of data privacy

To ensure confidentiality, invitees’ replies with contact information were separated from pseudonymized questionnaires. In order to protect the focus group participants’ identity, all names were replaced by numbers and all potentially identifying details were changed during the transcription of the recordings.

### Transcription of focus group recordings

All focus groups recordings were transcribed verbatim by a trained student research assistant following designated transcription rules. Transcripts were checked for accuracy by NJP.

### Data analysis

The transcripts were analyzed using structuring qualitative content analysis [44–46]. Qualitative content analysis extracts and preserves the essential content of the data, while significantly reducing the amount of data to be processed. It allows the integration of previous theoretical knowledge while being open to new findings. Deductive categories were derived using the review conducted by Murray et al. [47], other relevant studies (e.g. [6, 36, 48]) and the focus group guideline. However, due to the exploratory character of the study, a further focus was placed on inductive category development by summarizing content analysis [45] derived from the material to ensure the openness of the analysis. Since the guideline pretest only resulted in minor changes to the focus group guideline and the pilot group’s participants did not differ significantly from main study’s participants (except for in their area of registration) we decided to analyze all transcripts using the same category system.

Both researchers (CJ/NJP) read all transcripts to ensure familiarity with the material and discussed their first impressions of the material. The transcripts were broken down into fragments of analysis, which were coded using deductively and inductively formed categories. These fragments could adopt different sizes ranging from part of a sentence to one or more paragraphs (according to the amount of data needed to understand the content and context of the relevant fragment). The transcripts were coded by CJ and NJP separately and the results were discussed after coding each transcript. All categories and codes were described extensively in code-memos which were supported by typical quotes. To ensure intersubjective reproducibility and comprehensibility, the results were presented to and discussed with the interdisciplinary workgroup “qualitative methods” (led by NJP) of the Department of General Practice / Primary Care and the co-authors (BL – professor, psychological therapist, MD; TZ and ML – trained psychologists/postdoctorate researchers and MS – professor, MD). Then, the entire material was subjected to a second round of coding. Data was managed using MAXQDA 11 (Verbi GmbH).

## Results

### Participants

All in all, 42 general practitioners participated in six focus groups.

#### Sample (pilot group)

We conducted one focus group with general practitioners to pilot the interview guideline and discuss further questions to be posed during the main part of the study. This focus group took place in September 2015 with seven attendees (female = 2, male = 5) of the 49th Congress of the German College of General Practitioners and Family Physicians. For more information about the participants see Table 1.

**Table 1:** Participant characteristics of the pilot group

Sex	Years of practice	N
Female	< 10	2
	> 10	0
Male	< 10	1
	> 10	4

#### Sample (main study)

Five focus groups with a total of 35 participants (4–9 participants, female = 16, male = 19) were conducted between December 2015 and February 2016 in Hamburg. Four groups consisted of GPs without further training in psychosomatic medicine, while the last group also included GPs with specialized training in psychosomatic medicine. All participants except three had additional qualifications in basic psychosomatic care (“Psychosomatische Grundversorgung”). For more information about the participants see Table 2.

**Table 2:** Participant characteristics of the main study

Sex	Years of practice	Location of practice	N
Female	< 10	Hamburg	4
		Adjacent county	1
	> 10	Hamburg	8
		Adjacent county	3
Male	< 10	Hamburg	0
		Adjacent county	5
	> 10	Hamburg	7
		Adjacent county	7

### Main categories

Three main categories turned out to be relevant concerning GPs’ views on and approaches to coding and reasons for not recording MUS and somatoform disorders: benefits of coding, reasons for restrained coding and inaccurate coding. Table 3 gives an overview of the coded main and subcategories.

**Table 3:** Coding tree - main and sub categories

Main category	Subcategories
Benefits of coding	*Coding for reimbursement purposes* vs. *“inofficial” documentation*
	*Coding and (intended) therapy*
Inaccurate coding	*Non-stigmatizing diagnoses*
	*Tentative diagnoses and functional coding* vs. *confirmed diagnoses*
Reasons for restrained coding	–

#### Benefits of coding

##### Coding for reimbursement purposes vs. “inofficial” documentation

General practitioners have to document an ICD-10-GM code for every consultation with a patient in order to be reimbursed for their services by the statutory health insurance according to a set fee for certain services. Almost all interviewed GPs did not to use ICD-10-GM codes to document reasons for encounters or diagnoses in their informal reference files. They use ICD-10-GM only because they need to do so for reimbursement purposes.

*“Well, documentation is multifaceted. […] and it is also true that we are reimbursed for psychological codes, which means that I have to document an F-category diagnosis so that I have a billing code. […]”* (group F, paragraph 146).

*“I would write something along the lines of ‘all physical origins ruled out. Question psychological?’ For myself. Just to know that it was that point in time where I mentioned the possibility that it could be so. […]”* (group E, paragraph 97).

GPs mentioned that there are substantial differences between their ICD-10-GM-coding for billing purposes and their personal informal documentation (in the reference file) about symptoms presented and information given by the patient, for example about the psychosocial context or potential causes. The informal documentation is relevant for treatment purposes while the official documentation is more or less irrelevant for the individual consultation with the patient.

*“Just to clarify, after every consultation we have to document an ICD code, we have to, are forced to. […] What we write in our, well mostly no longer papers but what we write in our texts are the important facts or what we registered while taking the patient’s history. It doesn’t have to have anything to do with what we have to document. […]” (group A, paragraph 276).*

*“Unfortunately we often search desperately for a fitting cipher [ICD code]. […] We document ten codes which circumscribe the symptoms and somehow unclearly define the symptoms. In the end the main problem which we write or type into our private documentation is not coded. In that sense it’s one of the hundreds of additional jobs we have.”* (group B, paragraph 363).

##### Coding and (intended) therapy

Some GPs stated that they seldom used F-category diagnoses (chapter F: mental and behavioural disorders) from the ICD-10-GM, even if those diagnoses would be fitting for the patient’s condition. They only used them if a referral for psychotherapy was intended or if they wanted to account for longer consultations to explore psychosomatic symptoms.

“*[…] well, if I don’t refer a patient to psychosomatic therapy or psychotherapy, I don’t see the necessity in coding a F-category diagnosis. It’s basically sufficient to code back pain or headaches.*” (group A, paragraph 251).

“*There are two influencing factors. The one is the doctors’ fee scale [Einheitlicher Bewertungsmaßstab]. So, for example, if I have a patient with unclear bodily symptoms whom I don’t refer to psychotherapy or psychosomatic therapy, then that patient is coded for back pain or headaches. […] However, if, for example, I do a probationary session for short-term psychotherapy I definitely have to code an F-category diagnosis. […] or if bodily symptoms are the main problem then the code 45.1 somatisation disorder right?*” (group A, paragraph 288).

#### Reasons for restrained coding

##### Non-stigmatizing diagnoses

General practitioners are inclined to utilize diagnoses which they feel to be less stigmatizing or easier to accept from the patient’s point of view. Such diagnoses, for example fibromyalgia, tend to be more widely accepted than other diagnoses focusing on psychological aspects, or, for example, diagnosing an adjustment disorder seen as a temporary state rather than a depression. Another aspect is the perception that diagnoses of somatoform disorders (and other diagnoses from chapter F of the ICD-10-GM) stigmatize patients and lead to negative social and economic consequences concerning health, occupational disablement and life insurances.

“*[…], well the F-category diagnoses are very stigmatising and related to several significant disadvantages. If one codes an F-category diagnosis, so a confirmed somatoform disorder surely isn’t a big problem, but if one simply codes a depression then the patient won’t receive a credit or be able to switch to another health insurance if he is privately insured for the next 5 years. In statutory health insurance this isn’t a problem but one has to be very careful. I believe that somatisation disorders are not seen as gravely, but in other cases it’s a huge problem to just simply code such a diagnosis. […]”* (group D, paragraph 201).

“*[…] What surprises me is that no other diagnosis is coded. One that I would have expected with this clinical constellation is fibromyalgia. That a rheumatologist or an orthopedist codes such a diagnosis. What a lot of patients primarily consider relieving, especially when it comes to not letting the psychological aspects come so close. Fibromyalgia is purely a collection of particular phaenomena, a syndrome, but many patients view it as an illness. It is a relief for them to know ‘Someone labelled me. I feel taken seriously.’ Then these patients proceed to peddle through the healthcare system. […] More so than with the concept of a ‘somatisation disorder’.*” (group A, paragraph 99–101).

##### Tentative diagnoses and functional coding vs. confirmed diagnoses

GPs preferred symptom-oriented or functional coding rather than confirmed diagnoses for patients with MUS. For managing or treating these patients they do not necessarily need to document a confirmed diagnosis. Even to grant someone sick leave, an ICD-10-GM code describing functional symptoms is enough. Some GPs mentioned that – being a diagnosis of exclusion – somatoform disorders could never be a confirmed diagnosis in its proper sense.

“*I always document unspecific primarily somatic diagnoses on sick leave notes. That is the reason. I prefer not to confirm a specific diagnosis. That has a reason. I don’t confirm a diagnosis, I don’t have to. […]* ”(group B, paragraph 150).

*“Well, the question is, […]/ do I code the diagnosis. If I write or if I have to code the diagnosis in the program in my practice then I would of course document something similar but as a tentative diagnosis, you know? It obviously is never a confirmed diagnosis, right? […]* ”*(*group A, paragraph 110).

#### Inaccurate coding

General practitioners reported to be insufficiently informed about diagnostic criteria for somatoform disorders. Diagnoses are often recorded by choosing the first diagnosis fitting the patient’s symptoms, complaints or medical condition “by rule of thumb”. More general diagnoses are preferred to detailed ICD-10-codes. Finding the definite diagnosis is seen as the responsibility of psychiatric specialists not general practitioners. Time constraints are often mentioned as a reason for superficial or inaccurate coding.

*“I don’t know the criteria. […] I only know the ICD list of diagnoses, then I open the thing and take a look at what fits best. However, I have not taken an in depth look at coding psychosomatic disorders thus far.”* (group F, paragraph 182–184).

“*Well, I consider this extreme differentiation between a somatisation disorder or an adaptation disorder, that one has to go into so much detail, is something that the psychotherapists should do. […]*” (group E, paragraph 339).

“*I was totally annoyed when I received this flyer from the KV [Association of Statutory Insurance Health Physicians]: ‘You bill for psychosomatic diseases, here is a short summary for you.’ It was something, excuse me for saying so, that was a large as this piece of paper, folded several times and I thought: “Shit, so many diagnoses. I don’t have time for that”. So I apologize, but I used broad diagnoses because I believe that specific diagnoses should be made by specialists, I don’t have the time for it.*” (group D, paragraph 379).

*“Why should I do that? Why? I know what the patient’s problem is, I documented it, I look for a somewhat fitting diagnoses and am happy if I find one quickly and am done.*” (group C, paragraph 425).

## Discussion

### Main findings

Three main categories turned out to be relevant concerning GPs’ views on and approaches to coding and reasons for non-coding of MUS and somatoform disorders: benefits of coding, reasons for restrained coding and inaccurate coding. GPs reported coding for reimbursement purposes and using another language in their reference files for the patients. F-diagnoses from the ICD-10-GM were often only used if a referral for psychotherapy was intended or if they wanted to account for longer consultations to explore psychosomatic symptoms. General practitioners are inclined to utilize diagnostic codes which they feel to be less stigmatizing and not leading to negative social and economic consequences. GPs also felt insufficiently informed about the diagnostic criteria for somatoform disorders and to prefer more general diagnoses of the ICD-10-GM mentioning time constraints as a reason for superficial or inaccurate coding.

### Strengths and weaknesses

Reasons for not recording MUS/somatoform disorders have not been examined comprehensively before, documented by a lack of studies on (German) GPs’ coding behavior especially in respect to MUS and somatoform disorders. Many quantitative studies [16, 24, 29, 30, 39] state that problems regarding morbidity coding exist and make educated guesses about the reasons, but explorative qualitative studies were needed to shed light on this area. To our knowledge this is the first qualitative study (in Germany) to explore this theme.

Participation in our study was voluntary, which could have resulted in a selection of participants which are exceptionally interested in or burdened by the management and treatment of patients with MUS or somatoform disorders. We have no opportunity to rule out a possible (self-) selection bias, but the variation of accounts by the GPs indicates that the participants differed in their views on barriers and preferences on how to handle those patients. We also tried to maximize the variation by selecting participants according to age, gender and years of registration to further stimulate discussions [49]. Most focus group participants were licensed in Hamburg and adjacent counties meaning that their accounts represent the situation in northern Germany, however, differences between the results from these focus groups and the pilot group with GPs from all over Germany were negligible.

### Findings relative to other studies

Our findings shed light on the process of coding of MUS and somatoform disorders by German GPs. Although there seem to be problematic areas, GPs did not seem to contest the patients’ suffering and accepted uncertainty of diagnoses as elementary part of their work (see also [50]). Not all patients with MUS will or must receive a “double-coding” with a functional and mental disorder by their GPs and this might be absolutely adequate, as we know from our results that they receive treatment for their symptoms either way. Besides that, our data substantiate a view that GPs do code more severe forms of MUS and somatoform disorders, especially when GPs will further refer their patients to a (specialized) therapy.

Problems concerning the reliability and validity of documented diagnoses were also reported in several other studies, including the under- and over-reporting of diagnoses [51, 52], incongruences between the reason for consultation in the reference files and the diagnoses for reimbursement [24, 53] and use of “billing diagnoses” [24]. Löwe et al. reported a [18] limited criterion validity of somatoform disorder diagnoses in DSM-IV and ICD-10. Our interviewees assigned ICD-10-GM chapter F diagnoses (mental and behavioural disorders), if they recommend psychotherapy or for reimbursement purposes when consultations last longer than usual and require in-depth evaluation of psychosocial factors. There is some evidence that MUS and other mental health problems are eminent areas of under- and over-reporting: Erler et al. [53] showed that psychosocial reasons for consultations and symptoms with nonspecific aetiology like abdominal pain or vertigo were under-reported in routine data compared to the GPs’ reference files on patients, while mental health problems (chapter F-diagnoses) seem to be over-reported.

Studies showed that consultations with a high level of uncertainty in which no specific diagnosis can be established (as it is often the case with MUS or somatoform disorders) are burdensome for GPs and elicit both, discomfort and the fear of missing a rare or serious disease [50]. Consultations with a high level of uncertainty also lasted longer than other types of consultations [1]. Our focus group participants also reported time constraints, which might be even more intense after unusually long consultations. The uncertainty of coding a diagnosis of exclusion results in reluctance to do so.

The ICD-10-GM used in Germany and Austria is a very detailed classification system (e.g. with 13.097 classes for the ICD-10-GM 2005) and includes complicated coding rules [54]. GPs function as gatekeepers in the German health care system. They are treating an unselected patient population often having unspecific symptoms of unclear aetiology, the complex manageability of the ICD-10-GM is an important drawback on using it [55]. GPs do not only have to deal with codes for a single specialty, such as cardiologists or psychiatrists, but with more or less all possible codes. On the other hand, the consistent (ICD-) coding is needed as a common language for GPs, other specialists, clinicians and other parties of the health care system. Wockenfuss et al. [29] showed that the ICD- 10 can be reliably used for coding on a chapter level, while three- or four-digit-coding (as it is necessary in the German health care system) leads to high coding uncertainties in general practice. Generally, the error level rises with increasing coding refinement [56]. Our data points to an interesting detail: Although GPs might not code the adequate diagnoses on a three- or four-digit-level of the ICD-10-GM, they often reported to code at least some diagnoses from the chapter F.3 or F.4 in patients with MUS or somatoform disorders, leaving the more refined classification of the patient’s condition to the specialists of the field concerned. In doing so, GPs acknowledge that a certain proportion of the patient’s complaints appear to be genuinely psychosomatic. This may be due to the higher acceptability of these disorders in physicians and patients [34] or due to the syndrome overlap between depression, anxiety and somatization [5]. A similar phenomenon of inaccurate coding occurred in a study with routine data from German GPs, Erler et al. [53] showed that this data allows for a relatively reliable conclusion whether a patient has diabetes, but not which type of diabetes.

Patients with MUS or somatoform disorders feel offended if their symptoms are not primarily seen as of somatic origin [57] and have a high fear of being stigmatized [58]. By not focusing on the symptoms being medically unexplained, the new DSM-5 diagnostic category ‘somatic symptom disorder’ might reduce the stigma felt by patients. Our data shows that GPs respect those feelings and can also relate to them. Focus group participants tried to avoid giving patients diagnoses, which they and the patients associate with social stigmas and tried to consider potential consequences of recording psychiatric diagnoses. This is also true in Stone’s [50] study - where GPs applied diagnoses of somatoform disorders very cautiously – and for other psychiatric diagnoses [59] such as depression [60]. This is in line with the finding that the most accurate diagnosis might not always be the most helpful to the patient [50].

## Conclusions

Our results are based on participants’ accounts of their clinical reasoning and behavior in daily practice. It cannot be ruled out that direct observation of consultations and coding behavior would highlight different aspects of coding of MUS or somatoform disorders and unearth other practices. Further studies (e.g., participatory observation in situ) are needed to enrich the understanding of this phenomenon.

Researchers in health services research often rely on administrative data, for example for grouping patients by their diagnoses, to study patterns and outcomes of disease or to study variations in access to care, and quality, cost and effectiveness of care [61]. The abovementioned studies and our findings supply qualitative evidence for the commonly seen difference between routine and epidemiological data [62]. Routine data created for billing or reimbursement purposes respectively, should be used with restraint and prudence [24, 27, 55, 62]. For researchers this results in a need to validate routine diagnostic data where possible [27].

From GPs’ points of view, ICD-10-coding does not seem to be a necessary requirement for treating patients (except with psychotherapy) and coding might be avoided to protect the patient from stigmas and other negative consequences. Our insights on GPs’ coding behavior do not allow conclusions about documentation, diagnostic and treatment quality.

The changes in the DSM-5 and the upcoming changes in the ICD-11 will bring the era of somatoform disorders - a diagnoses of exclusion - to an end [26]. The term ‘somatoform disorder’ is changed to ‘somatic symptom disorder’ in the DSM-5. Now, the diagnosis can be considered by the GP as one single somatic symptom is present. The distinction between medically explained and unexplained symptoms has been given up (criterion A). The B criterion (‘positive psychological criteria’) requires one or more of the following to apply: health anxiety or disproportionate and persistent concern about the medical seriousness of one’s symptoms respectively excessive time and energy to be devoted to these symptoms or health concerns [26]. Considering the already existing beta version of the ICD-11 one can assume that the ICD-11 will adopt these changes, even though it is not finally consented yet.

The described developments of substituting diagnoses of exclusion by the use of positive psychological criteria will supposedly change acceptance and handling of these diagnoses for both parties, GPs and patients. Nevertheless, the problem of “double-coding” and the abovementioned general unattractiveness of the ICD-system for many GPs due to its complexity will remain. Therefore the consequences of these changes for GPs’ diagnosing and coding behavior are not yet foreseeable. DSM-5’s somatic symptom disorder might be a concept that is easier applicable in primary care, whilst it is still challenged by implementation issues and the consequences of exact diagnostic coding of psychological disorders in this setting.

## Additional file

Additional file 1: Focus group guideline (Project: “Identification of barriers and difficulties involved in the process of diagnosing somatic symptom disorders in primary care”). (PDF 179 kb)

## Abbreviations

DEGAM: Deutsche Gesellschaft für Allgemeinmedizin und Familienmedizin (German College of General Practitioners and Family Physicians)
DSM: Diagnostic and Statistical Manual of Mental Disorders
GP: General practitioner
ICD-10: International Classification of Diseases, 10. Revision
ICD-10-GM: International Classification of Diseases, 10. Revision, German Modification
ICPC-2 PLUS: International Classification of Primary Care, Version 2 PLUS
MUS: Medically unexplained symptoms

## References

[1] Rosendal M, Carlsen AH, Rask MT, Moth G (2015). Symptoms as the main problem in primary care: a cross-sectional study of frequency and characteristics. Scand J Prim Health Care.

[2] Tschudi-Madsen H, Kjeldsberg M, Natvig B, Ihlebaek C, Straand J, Bruusgaard D (2014). Medically unexplained conditions considered by patients in general practice. Fam Pract.

[3] den Boeft M, van der Wouden JC, Rydell-Lexmond TR, de Wit NJ, van der Horst HE, Numans ME (2014). Identifying patients with medically unexplained physical symptoms in electronic medical records in primary care: a validation study. BMC Fam Pract.

[4] Steinbrecher N, Koerber S, Frieser D, Hiller W (2011). The prevalence of medically unexplained symptoms in primary care. Psychosomatics.

[5] Löwe B, Spitzer RL, Williams JBW, Mussell M, Schellberg D, Kroenke K (2008). Depression, anxiety and somatization in primary care: syndrome overlap and functional impairment. Gen Hosp Psychiatry.

[6] Fink P, Schröder A (2010). One single diagnosis, bodily distress syndrome, succeeded to capture 10 diagnostic categories of functional somatic syndromes and somatoform disorders. J Psychosom Res.

[7] Creed F, Guthrie E, Fink P, Henningsen P, Rief W, Sharpe M (2010). Is there a better term than “medically unexplained symptoms”?. J Psychosom Res.

[8] Kroenke K, Sharpe M, Sykes R (2007). Revising the classification of somatoform disorders: key questions and preliminary recommendations. Psychosomatics.

[9] Marks EM, Hunter MS (2015). Medically unexplained symptoms: an acceptable term?. Br J Pain.

[10] World Health Organisation (1992). International statistical classification of diseases and related health problems, 10th revision (ICD-10).

[11] American Psychiatric Association (2000). Diagnostic and statistical manual of mental disorders: DSM-IV-TR.

[12] Van den Bergh O, Witthöft M, Petersen S, Brown RJ (2017). Symptoms and the body: taking the inferential leap. Neurosci Biobehav Rev.

[13] Voigt K, Nagel A, Meyer B, Langs G, Braukhaus C, Löwe B (2010). Towards positive diagnostic criteria: a systematic review of somatoform disorder diagnoses and suggestions for future classification. J Psychosom Res.

[14] Fink P, Rosendal M, Olesen F (2005). Classification of somatization and functional somatic symptoms in primary care. Aust N Z J Psychiatry.

[15] Wessely S, Nimnuan C, Sharpe M (1999). Functional somatic syndromes: one or many?. Lancet Lond Engl.

[16] Noll-Hussong M, Otti A (2015). Functional and somatoform disorders in the mirror of ICD-10 routine data. Psychother Psychosom Med Psychol.

[17] Rosendal M, Vedsted P, Christensen KS, Moth G (2013). Psychological and social problems in primary care patients - general practitioners’ assessment and classification. Scand J Prim Health Care.

[18] Löwe B, Mundt C, Herzog W, Brunner R, Backenstrass M, Kronmüller K (2008). Validity of current somatoform disorder diagnoses: perspectives for classification in DSM-V and ICD-11. Psychopathology.

[19] Office P. ICPC-2 PLUS overview [Internet]. http://sydney.edu.au/medicine/fmrc/icpc-2-plus/index.php. Accessed 24 Oct 2017.

[20] Jensen-Doss A, Weisz JR (2008). Diagnostic agreement predicts treatment process and outcomes in youth mental health clinics. J Consult Clin Psychol.

[21] Swinglehurst D, Greenhalgh T (2015). Caring for the patient, caring for the record: an ethnographic study of “back office” work in upholding quality of care in general practice. BMC Health Serv Res.

[22] Majeed A, Car J, Sheikh A (2008). Accuracy and completeness of electronic patient records in primary care. Fam Pract.

[23] Choices NHS. Your health records - The NHS in England - NHS Choices 2016. http://www.nhs.uk/nhsengland/thenhs/records/healthrecords/pages/overview.aspx. Accessed 24 Oct 2017.

[24] Zimmermann T, Kaduszkiewicz H, vd Bussche H, Schön G, Wegscheider K, Werle J (2012). Reliability of morbidity data reported by GPs. Results of a longitudinal study in primary care. Bundesgesundheitsblatt Gesundheitsforschung Gesundheitsschutz..

[25] Cartwright DJ (2013). ICD-9-CM to ICD-10-CM codes: what? Why? How?. Adv Wound Care.

[26] Rief W, Martin A (2014). How to use the new DSM-5 somatic symptom disorder diagnosis in research and practice: a critical evaluation and a proposal for modifications. Annu Rev Clin Psychol.

[27] Davis KAS, Sudlow CLM, Hotopf M (2016). Can mental health diagnoses in administrative data be used for research? A systematic review of the accuracy of routinely collected diagnoses. BMC Psychiatry.

[28] Nilsson G, Petersson H, Ahlfeldt H, Strender LE (2000). Evaluation of three Swedish ICD-10 primary care versions: reliability and ease of use in diagnostic coding. Methods Inf Med.

[29] Wockenfuss R, Frese T, Herrmann K, Claussnitzer M, Sandholzer H (2009). Three- and four-digit ICD-10 is not a reliable classification system in primary care. Scand J Prim Health Care.

[30] Münch C, Gottschall M, Hübsch G, Köberlein-Neu J, Schübel J, Bergmann A (2016). Qualität der hausärztlichen Diagnosedokumentation in Patientenakten – Eine Analyse am Beispiel von Schilddrüsenerkrankungen. Z Für Evidenz Fortbild Qual Im Gesundheitswesen.

[31] Levenson JL (2011). The somatoform disorders: 6 characters in search of an author. Psychiatr Clin North Am.

[32] Deutsches Institut für Medizinische Dokumentation und Information (DIMDI). [International Statistical Classification of Diseases and Related Health Problems, 10. Revision, German Modification]. 2017. https://www.dimdi.de/dynamic/de/klassi/downloadcenter/icd-10-gm/. Accessed 24 Oct 2017.

[33] de Lusignan S, Minmagh C, Kennedy J, Zeimet M, Bommezijn H, Bryant J (2001). A survey to identify the clinical coding and classification systems currently in use across Europe. Stud Health Technol Inform.

[34] Hamilton JC, Eger M, Razzak S, Feldman MD, Hallmark N, Cheek S (2013). Somatoform, factitious, and related diagnoses in the national hospital discharge survey: addressing the proposed DSM-5 revision. Psychosomatics.

[35] Burton C, McGorm K, Richardson G, Weller D, Sharpe M (2012). Healthcare costs incurred by patients repeatedly referred to secondary medical care with medically unexplained symptoms: a cost of illness study. J Psychosom Res.

[36] Scobie S, Basnett I, McCartney P (1995). Can general practice data be used for needs assessment and health care planning in an inner-London district?. J Public Health Med.

[37] Nilsson G, Ahlfeldt H, Strender L-E (2002). Computerisation, coding, data retrieval and related attitudes among Swedish general practitioners-a survey of necessary conditions for a database of diseases and health problems. Int J Med Inf..

[38] Freidl M, Piralic-Spitzl S, Grohe N, Aigner M (2012). Association between fear of stigma, depressive and anxiety symptoms in patients with somatoform pain disorder. Psychiatr Prax.

[39] Schaefert R, Laux G, Kaufmann C, Schellberg D, Bölter R, Szecsenyi J (2010). Diagnosing somatisation disorder (P75) in routine general practice using the international classification of primary care. J Psychosom Res.

[40] Heinbokel C, Lehmann M, Pohontsch NJ, Zimmermann T, Althaus A, Scherer M (2017). Diagnostic barriers for somatic symptom disorders in primary care: study protocol for a mixed methods study in Germany. BMJ Open.

[41] Marshall MN (1996). Sampling for qualitative research. Fam Pract.

[42] Finch J (1987). The vignette technique in survey research. Sociology.

[43] Barter C, Renold E. Social Research Update 25: The Use of Vignettes in Qualitative Research. http://sru.soc.surrey.ac.uk/SRU25.html. Accessed 24 Oct 2017.

[44] Dey I (1993). Qualitative data analysis: a user-friendly guide for social scientists.

[45] Schreier M. Ways of doing qualitative content analysis: disentangling terms and terminologies. Forum Qual Soc Res. 2014; http://www.qualitative-research.net/index.php/fqs/article/view/2043. Accessed 24 Oct 2017

[46] Hsieh H-F, Shannon SE (2005). Three approaches to qualitative content analysis. Qual Health Res.

[47] Murray AM, Toussaint A, Althaus A, Löwe B (2016). The challenge of diagnosing non-specific, functional, and somatoform disorders: a systematic review of barriers to diagnosis in primary care. J Psychosom Res.

[48] de Lusignan S, Wells SE, Hague NJ, Thiru K (2003). Managers see the problems associated with coding clinical data as a technical issue whilst clinicians also see cultural barriers. Methods Inf Med.

[49] Przyborski A (2014). Qualitative social research: a workbook.

[50] Stone L (2013). Making sense of medically unexplained symptoms in general practice: a grounded theory study. Ment Health Fam Med.

[51] Brenner G, Koch H, Kerek-Bodden H, Heuer J, Lang A, Lang A (2007). Diagnoses as the subject of health service research to analyse the morbidity of outpatients. Bundesgesundheitsblatt Gesundheitsforschung Gesundheitsschutz.

[52] Gerste B, Gutschmidt S. Quality of diagnostic data from outpatient care - Critical notes using the example of diabetes. Gesundheits Soziopol. 60(3–4):29–43.

[53] Erler A, Beyer M, Muth C, Gerlach FM, Brennecke R (2009). Garbage in - garbage out? Validity of coded diagnoses from GP claims records. Gesundheitswesen Bundesverb Arzte Offentlichen Gesundheitsdienstes Ger.

[54] Kassenärztliche Bundesvereinigung. Wegweiser ICD-10. http://www.kbv.de/html/2007.php. Accessed 24 Oct 2017.

[55] Stausberg J, Lehmann N, Kaczmarek D, Stein M (2005). Uniform coding in Germany: wish and reality. Krankenh..

[56] Stausberg J, Lehmann N, Kaczmarek D, Stein M (2008). Reliability of diagnoses coding with ICD-10. Int J Med Inf.

[57] Stone J, Wojcik W, Durrance D, Carson A, Lewis S, MacKenzie L (2002). What should we say to patients with symptoms unexplained by disease? The “number needed to offend”. BMJ.

[58] Freidl M, Spitzl SP, Prause W, Zimprich F, Lehner-Baumgartner E, Baumgartner C (2007). The stigma of mental illness: anticipation and attitudes among patients with epileptic, dissociative or somatoform pain disorder. Int Rev Psychiatry Abingdon Engl.

[59] Kruse J, Schmitz N, Wöller W (2004). Warum übersieht der Hausarzt die psychischen Störungen seiner Patienten? : Determinanten der hausärztlichen Identifikation psychischer Störungen. Psychother Psychosom Med Psychol.

[60] Sielk M, Abholz HH (2005). Why do general practitioners characterize other patients as depressive than psychiatrists do?. Z Allg Med..

[61] O’Malley KJ, Cook KF, Price MD, Wildes KR, Hurdle JF, Ashton CM (2005). Measuring diagnoses: ICD code accuracy. Health Serv Res.

[62] Kreis IA, Busby A, Leonardi G, Meara J, Murray V (2013). Essentials of environmental epidemiology for health protection: a handbook for field professionals.

