# Supplementary material for: Coding of medically unexplained symptoms and somatoform disorders by general practitioners – an exploratory focus group study
Source: BMC Fam Pract. 2018 Jul 27;19:129. doi: 10.1186/s12875-018-0812-8 (PMC6064152; doi:10.1186/s12875-018-0812-8)
Supplement: Supplementary file 1 — Focus group guideline (Project: “Identification of barriers and difficulties involved in the process of diagnosing somatic symptom disorders in primary care”). (PDF 179 kb) [file 12875_2018_812_MOESM1_ESM.pdf]

Focus group guideline (Project: “Identification of barriers and difficulties involved in the process of diagnosing somatic symptom disorders in primary care”)

A) Presentation of the first part of the vignette
A1) Which problems/difficulties do you face at this point?<br>A2) What would you do with this patient?
B) Presentation of the second part of the vignette
B1) Which problems/difficulties do you face now?<br>B2) What impedes diagnosing a somatoform disorder for this patient?<br>B3) How do you recognize such a disorder?<br>B4) How do you document such a disorder?<br>B5) How do you communicate your (suspected) diagnosis to the patient?<br>B6) Which feelings and behaviors does the patient’s behavior elicit in you?
C) Empirical studies name different possible consequences of a delayed diagnosis of somatoform disorders: unnecessary somatic diagnostics, delayed start of treatment, iatrogenic harm, e.g. through unnecessary invasive diagnostics or operations. Being a general practitioner you have an important function in the management of these patients. Therefore we are interested to hear your thoughts on the following aspects:<br>C1) How do you identify a patient having a somatoform disorder?<br>C2) Which aids do you use to identify these patients?<br>C3) What would help you to diagnose a somatoform disorder?<br>C4) How do the ICD-10 criteria influence your daily practice with these patients?
